# Low-Cost Microcontroller-Based Multiparametric Probe for Coastal Area Monitoring

**DOI:** 10.3390/s23041871

**Published:** 2023-02-07

**Authors:** Lorena Parra, Sandra Viciano-Tudela, David Carrasco, Sandra Sendra, Jaime Lloret

**Affiliations:** Instituto de Investigación para la Gestión Integrada de Zonas Costeras, Universitat Politècnica de València, C/Paranimf, 1, 46730 Valencia, Spain

**Keywords:** water quality, salinity, total dissolved solids, physical sensor, inductive coils, light abortion, electromagnetic sensor, optical sensor

## Abstract

The monitoring of the coastal environment is a crucial factor in ensuring its proper management. Nevertheless, existing monitoring technologies are limited due to their cost, temporal resolution, and maintenance needs. Therefore, limited data are available for coastal environments. In this paper, we present a low-cost multiparametric probe that can be deployed in coastal areas and integrated into a wireless sensor network to send data to a database. The multiparametric probe is composed of physical sensors capable of measuring water temperature, salinity, and total suspended solids (TSS). The node can store the data in an SD card or send them. A real-time clock is used to tag the data and to ensure data gathering every hour, putting the node in deep sleep mode in the meantime. The physical sensors for salinity and TSS are created for this probe and calibrated. The calibration results indicate that no effect of temperature is found for both sensors and no interference of salinity in the measuring of TSS or vice versa. The obtained calibration model for salinity is characterised by a correlation coefficient of 0.9 and a Mean Absolute Error (MAE) of 0.74 g/L. Meanwhile, different calibration models for TSS were obtained based on using different light wavelengths. The best case was using a simple regression model with blue light. The model is characterised by a correlation coefficient of 0.99 and an MAE of 12 mg/L. When both infrared and blue light are used to prevent the effect of different particle sizes, the determination coefficient of 0.98 and an MAE of 57 mg/L characterised the multiple regression model.

## 1. Introduction

The oceanic ecosystem covers about 70% of the Earth’s surface. It is estimated that the oceans contain around 97% of the total water on the Earth [1]. Oceans are known for their extraordinary biodiversity [2]. Exploiting these ecosystems has increased the environmental impact in recent decades. Moreover, the impact linked to activities linked to tourism in coastal areas has risen [3]. The study of coastal areas is challenging due to their high biodiversity and the wide variety of human activities developed in these locations [4]. Each habitat presents physicochemical and topological characteristics [5], including the distributions of nutrients, organisms, water quality, temperature, pH, and salinity [6]. The oceans present temporal variability, annual, seasonal and daily changes, which involve phenomena such as tides, river plumes, marine currents, weather, and maritime traffic, among others [7]. In addition, the exploitation of resources by human beings causes alterations in the ecosystem, occasionally leading to severe damage [8]. Therefore, monitoring the water quality in the oceans is crucial to better managing coastal and marine areas [9]. The study of water physicochemical parameters allows the application of smart green protocols to ensure the sustainability of the exploitation of resources, as well as the protection of biodiversity.

Remote sensing is the most widely used system for monitoring the land, oceans, and seas [10]. These systems are mainly based on obtaining information from the studied surfaces from the electromagnetic waves that are emitted [11]. Different information sources for remote sensing include satellites, aircraft, and drones. Satellites present different resolutions, and some have excellent spatial resolution that allows the obtaining of highly accurate values of the studied parameters [12]. Nevertheless, the temporal resolution is low, considering that the studied phenomenon usually presents changes in a short period. Therefore, satellites might not detect the change produced in the time necessary to act if necessary. Some examples of satellites used for land and oceans monitoring are Landsat [13], Sentinel [14], MODIS [15], and NOAA [16]. Moreover, satellites do not allow studies in sea depths or studies of the variation along the water column.

An alternative to remote sensing systems is the Wireless Sensor Network (WSN). Although WSNs might have a low spatial resolution, they are characterised by offering an excellent temporal resolution and the possibility of having real-time information [17]. The implementation of low-cost sensors and the reduction in communication technologies and deployment costs allow for increasing the number of nodes, enhancing the spatial resolution. These low-cost WSNs are currently used in many fields of study, such as agriculture, health, smart cities, and oceans [18]. Their use has increased in recent years for water quality monitoring in fresh and saltwater ecosystems. In addition to optical sensors (such as light-emitting diodes (LEDs) and photodiodes), electromagnetic sensors are used to measure parameters such as water conductivity [19].

Electromagnetic sensors are based on the external electromagnetic field near and inside the studied environment. This type of sensor can be implemented in many different cases. An example of this is the monitoring of the evolution of scour in the foundations of bridges, using intelligent probes based on electromagnetic sensors [20]. There are studies in which this type of sensor is used to detect water pollution by nitrates [21,22]. Another type of electromagnetic sensor is the inductive coil, which allows the detection of water pollution and uses the flow produced to apply Internet of Things sensors [23].

This work aims to design and test a sensor node based on electromagnetic sensors, such as inductive coils combined with optical sensors. In this way, it is intended to determine the water quality through parameters such as conductivity and turbidity in different aquatic environments. Conductivity is associated with total dissolved solids (TDS), which are associated with turbidity and Total Suspended Solids (TSS), thus allowing the detection of the discharge of pollutants in the coastal areas. The calibration test is composed of up to 25 calibration samples with variable values of salinity and TSS. Compared with previous studies regarding the nodes, the novelty of this study is the integration in a single device of existing commercial and own-developed optical and electromagnetic sensors and the analysis of its operation. In addition, previous sensors were calibrated for conductivity and turbidity; in this case, the calibration will be for salinity and TSS. The paper presents the design, creation and calibration of the sensor node for the water quality monitoring of coastal areas. The main contributions are the following:

- The complete design of the low-cost node based on physical sensors to measure physical and chemical parameters is presented. We include the creation of new physical sensors, which can be easily adapted to commercial devices due to their low cost and good sensibility. In addition, the design of the node, including the software and hardware aspects, is described.
- Regarding the measurements, we have studied the possible interferences in the performance of the sensor, including the following: (i) electrical interferences of sensors’ signal and circuits for powering the sensor and receiving the signal, (ii) interference of salinity in the optical sensor for TSS measurement, (iii) interference of TSS in the electromagnetic sensor for salinity measurement, and (iv) interference of temperature on both TSS and salinity sensor.

The rest of the paper is structured as follows: Section 2 outlines different studies related to the determination of water quality, such as conductivity and turbidity. Section 3 describes how the development of the proposed system has been carried out. The test bench for the calibration is detailed in Section 4. The results are discussed in Section 5. Finally, Section 6 summarises the conclusion and future work.

## 2. Related Work

This section shows different studies on the implementation of sensors to determine water quality (conductivity and turbidity) in aquatic environments. In addition, studies related to remote sensing are presented as well as how different techniques have been interconnected to establish conductivity and turbidity values for decision-making.

Brinda Das and P.C. Jain, in 2017 [24], implemented a real-time water quality system using different sensors (pH, conductivity, temperature). They used the ZigBee module to transfer the data obtained to the microcontroller. The GSM module sends these data to a smartphone or computer. In addition, the system sends messages to alert them of discharges that may contaminate the water. In 2015, Parra et al. [25] developed a low-cost sensor based on conductivity. To do this, they used two coils using the method of mutual inductance. They tested five prototypes and concluded that the coils that gave the best results were those of 40 turns and 80 turns (fed and induced, respectively). With the results obtained, they extracted an equation that made it possible to calculate the water conductivity with high precision. Wang et al. in 2020 [26] reviewed conductivity systems. The sensors used were based on conductive polymers (CP). CPs are mainly composed of polyaniline, polypyrrole and poly(3,4-ethylene dioxythiophene). These were prepared under different polymerisation conditions and used as various sensors. It was observed that CP and other detection materials, such as metals, metal oxides, etc., show a high detection performance.

In addition to conductivity, another parameter which characterises water quality is turbidity. In 2016, Azman et al. [27] developed a nephelometric turbidity sensor for continuous water quality monitoring. The results obtained can be visualised directly by the consumer. They compared the developed sensor with the commercial turbidity sensor (Hach 2100P) from the Nephelometric Turbidity Unit (NTU). This system is based on the use of LEDs that transmit light and receivers based on light-dependent resistors. They communicated with the central processor (PIC 16F777) and a module (RS232). Arifin et al. in 2017 [28] developed a turbidity sensor of different wavelengths of polymer optic fibre to measure turbidity. They used infrared LED, photodetector and polymer optic fibre. As a result, they found that the output voltage is affected by the length of the sensor, whether or not it has a coating, the curvature of the sensor and the concentration of turbidity that the water presents.

In 2018, Wang et al. [29] developed a low-cost sensor for turbidity monitoring. The sensor is based on detecting transmitted and scattered light by using 850 nm (insert full name) infrared LEDs and dual orthogonal photodetectors. The proposed design can measure turbidity within the 0–1000 NTU range. Mulyana and Hakim [30], in the same year, developed Arduino Leonardo, a water turbidity monitoring system. They used a photodiode and an infrared LED to implement the turbidity sensor. They processed the analogue signal received by the Arduino Leonardo. They found that the turbidity detection range for the developed sensor was between 0 NTU and 40 NTU. Finally, Parra et al. [31] developed a turbidity system to differentiate the type of turbidity it was. The implemented sensor consists of four LEDs. In addition, it is composed of photodiodes and resistors, which are located at 180°. They used different types of samples; *Isochrysis galbana*, *Tetraselmis chuii* and sediment. Finally, they created an algorithm to establish the turbidity, the concentration of the turbidity source and the origin of it. As a result, they found that the type of turbidity present in the water can be differentiated.

Mansor et al. in 2022 [32] developed an alert system for water pollution in rivers. With the implementation of this system, they intend to notify the authorities when there is an anomaly. It is a low-cost system. The data obtained through ammoniacal nitrogen, suspended solids and biochemical oxygen demand sensors are sent via WiFi to the ThingSpeak platform. They found that the errors of the conductivity sensor were 6.84% and 6.35%, comparing it with the reference sensor. They finally obtained accurate readings for the turbidity sensor.

In contrast to the works presented so far, water quality and remote sensing studies are shown. In 2017, Sharaf El Din et al. [33] based their study on determining the deterioration of surface water. They mapped different surface water quality parameters (SWQP) to do this. Additionally, they developed an artificial intelligence modelling method to map this type of water based on the backpropagation neural network to quantify the concentrations of different parameters by using the images of the Landsat 8 satellite. They concluded that using that neural network based on Landsat8 can establish concentrations of different SWQPs from Landsat8 images. Abdelmalik, in 2018 [34], wanted to show a spatial distribution map in Lake Qaroun for each water quality parameter. He used data from Advanced Spaceborne Thermal Emission and Reflection Radiation (ASTER) to do this. Among the parameters analysed in this study are the electrical conductivity of water and turbidity, as well as temperature. He used different samples to obtain the relationship between the water quality parameters and the ASTER values. As a result, he found a significant correlation between the observed values and the remote sensing data, with R2 > 0.94 sig. < 0.01. Finally, in 2020, Sagan et al. [35] evaluated the ability of remote sensing to assess water quality. This study analysed more than 200 data sets, including dissolved oxygen, organic matter, and electrical conductivity. They combined the data taken in the laboratory and collected by proximal sensors distributed in different areas with hyperspectral images and satellite data. As a result, they found that the optically active parameters can be obtained by the satellites. However, those optically non-active parameters can be estimated indirectly, but there are still problems. To do this, you have to use deep learning.

Although all these studies present significant advances, they also offer some difficulties. For example, remote sensing often only allows for continuous monitoring of the study area. In addition, image processing techniques are often expensive and complicated to handle. However, the present study shows that combining different sensors for developing a low-cost parametric probe can allow the evaluation of different water quality parameters. With the evolved low-cost node, continuous conductivity monitoring is allowed, as well as turbidity determination. In this case, the conductivity is measured as salinity, which is also known as Total Dissolved Solids (TDS). Salinity or TDS is measured in g/L and is the summation of diluted solids in the water. Regarding turbidity, it is measured in terms of TSS, which represents the amount of solids in water which are not diluted or sedimented. The TSS is usually measured in mg/L. The proposed solution also allows more excellent coverage to be established due to the large number of sensors deployed throughout the study area. In addition, these temperature measurements can be compensated for by using a temperature sensor.

## 3. Overall Description of the Proposed Water Quality Monitoring System

This section describes the sensor node developed for water quality monitoring. The system is composed of a low-cost node capable of collecting and storing environmental data on an SD card.

### 3.1. Sensor Node

In order to develop the water quality sensor node, an Arduino Leonardo module is used [36]. Other microcontrollers can be used; we have selected the Arduino Leonardo module for its features and its quality–price rate. The Arduino Leonardo module is a board based on an Atmega328 microcontroller. Its features are shown below:

- Microcontroller: ATmega328
- Operating Voltage: 5 V
- Input Voltage (Recommended): 7–12 V
- Digital Input/Output Pins: 14 (6 of them are PWM outputs)
- Analog Input Pins: 6
- DC current per pin: 20 mA/pin
- DC current for 3.3 V output pin: 50 mA/pin
- Flash Memory: 32 KB (ATmega328) of which 0.5 KB is used by Bootloader
- SRAM: 2 KB (ATmega328)
- EEPROM: 1 KB (ATmega328)
- Clock Speed: 16 MHZ
- Physical characteristics: Weight (25 gr), Width (53.4 mm), Length (68.6 mm).

Likewise, different peripheral sensors useful in environmental monitoring are required. Specifically, the system is able to measure the levels of salinity, the amount of TSS and the water temperature which is used, if necessary, to compensate the measurements of salinity and TSS.

### 3.2. Water Salinity Sensor

**Figure 1:**
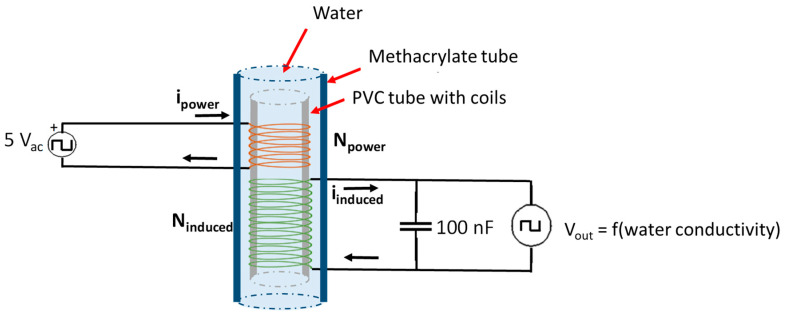
Diagram of our water salinity sensor.

The water salinity sensor is based on the use of two different coils coiled over a PVC tube and introduced into a water sample. As is shown in [25], the principle of operation of this sensor is based on the concept of mutual inductance between a powered coil ($L_{power}$) and an induced coil ($L_{induced}$). In this case, the sensor has two coils with lengths hp and hi and a number of spires: $N_{power}$ and $N_{induced}$. Additionally, the sensor does not have a ferromagnetic core. Instead of this, it has a cylindric space, section S, which will be filled with water with concentrations of dissolved salts with a relative permeability $\mu_{r\_water}$. The solenoids are coiled over the PVC pipe with a diameter of 2.6 cm. The wire used to form the coils is enamel copper wire with a calibre of 0.4 mm. For our deployment, $L_{power}$ has 40 spires while $L_{induced}$ is composed of 80 spires. Figure 1 shows the circuit used to create our water salinity sensor. As Figure 1 shows, the developed sensor is inserted inside the methacrylate tube, which is used to measure other parameters with the rest of the device. This sensor is an own design used previously as a conductivity meter.

Regarding the manner of feeding the powered coil, the system also contains a 7555 integrated circuit in Astable mode [37] to create a square signal that alternates between a positive voltage of 5 V and 0 V. Finally, the output voltage that comes from an induced coil is sent to a general-purpose diode and a low-pass filter to rectify the alternating signal and obtain a continuous value.

### 3.3. TSS Sensor

**Figure 2:**
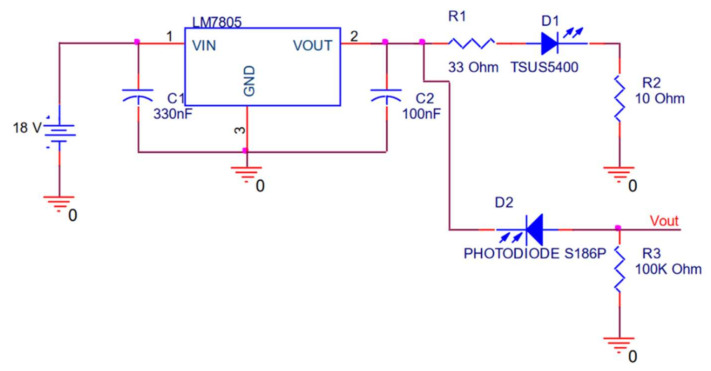
The electronic circuit of the turbidity sensor.

The water TSS sensor [38] is based on the use of an LED [39] and a photodiode [40] in the infrared band. The infrared LED and the photodiode are placed at a distance of 3.2 cm. Both elements work at 900 nm and are disposed at an angle of 180°. Both devices are supplied with a voltage of 5 V, which can be directly extracted from the Arduino module. An alternative to power this circuit is the use of a voltage regulator of LM7805, powered with a voltage up to 25 Volts, since this type of regulation permits a maximum output current of 1 A. Figure 2 shows the electronic circuit of our proposed system. The Vout terminal is connected to an analogue pin of the Arduino module. This sensor is an own design previously used for determining and characterising turbidity.

**Figure 3:**
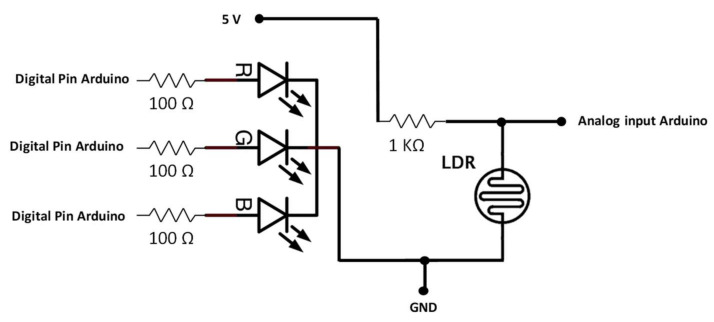
Schematic of RGB sensor.

In order to improve the TSS measuring and provide the sensor with further use, an RGB LED and an LDR are used. The sample of water is illuminated by the RGB LED, and the LDR registers the amount of light that reaches it. As Figure 3 shows, the RGB LED uses a common cathode connected to the GND, while terminals that control each colour are connected to an output pin of the Arduino module through a resistor between 100 Ω and 330 Ω. On the other hand, the LDR is powered by a 5 V Arduino pin, and the voltage value, which is proportional to the amount of received light, is registered by an analogue input of the Arduino module. Additionally, a resistor of 1 kΩ is added, creating a voltage divisor to better control the amount of voltage the Arduino receives.

### 3.4. Water Temperature Sensor

**Figure 4:**
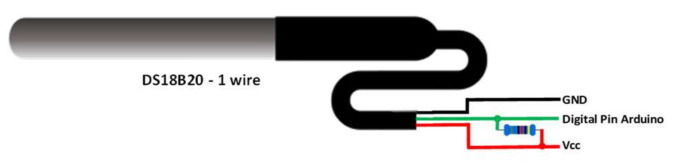
Connections for DS18B20.

The DS18B20 temperature sensor [41], a commercial probe, is one of the most versatile sensors available on the market. This sensor is suitable when the temperature in humid or water environments should be measured since there is a waterproof probe model. The DS18B20 can measure temperatures between −55 °C and 125 °C. It has a very wide range; however, it does not have the same error in the entire range. Specifically, for temperatures between −10 °C and 85 °C, the sensor has an error of ±0.5 °C, while for the rest of the temperatures between −55 °C and 125 °C the error is ±2 °C. Figure 4 shows the electric connections of this sensor to a digital pin of an Arduino.

### 3.5. Timing Tag of Measurements

**Figure 5:**
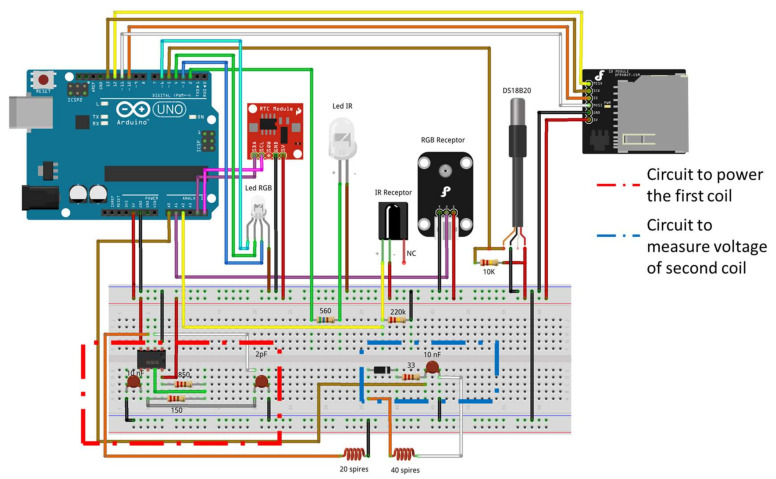
Full circuit of our sensor node.

**Figure 6:**
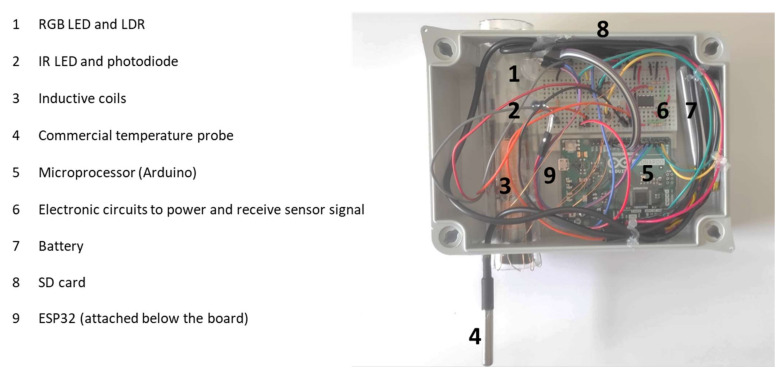
Real image of our prototype.

Finally, the measurements collected are tagged using a time tag provided by the real-time clock (RTC). There are several RTC models widely used in electronic developments, among which we can highlight the DS1307 [42] and the DS3231 [43]. The DS1307 model is highly affected by temperature variations. This fact affects the time measurement of the resonator crystals, translating it into errors in an accumulated lag, which can be 1 or 2 min per day. To solve this, the DS3231 incorporates a temperature measurement mechanism and compensation system that guarantees an accuracy of at least 2 ppm, which is equivalent to a maximum lag of 172 ms/day (1–2 s/month). Our system incorporates the RTC DS3231, which guarantees the correct operation for a longer time. Figure 5 shows the connection diagram of the entire system, while Figure 6 shows a real photo of the prototype of the system. The RTC is also used to generate a routine in the node to ensure data gathering every hour and to put the node into a deep sleep in the meantime to save energy. This enhances the battery life, allowing more extended monitoring periods.

### 3.6. Communication Technology

Concerning the communication of the node, there are different options for communicating information to the gateway and across the network. The most simple one, which is implemented for the tests conducted in this paper, is to integrate an ESP32 module that allows a WiFi connection with the Arduino Leonardo. To do this, we connect the Arduino Leonardo and ESP32 modules using the UART port.

## 4. Test Bench

In this section, the material and methods for the calibration test are defined. First, we describe the preparation of calibration samples, the materials used, the number of samples, and how they are generated. Next, the calibration procedure is defined.

### 4.1. Calibration Samples

Different samples were created to conduct a complete calibration and evaluate the performance of the different sensors included in the multiparametric probe; see Table 1. The samples were created according to the expected values in the coastal areas, which might differ from other marine areas. Generally, the salinity is lower and the turbidity is greater in the coastal area. Since, in our possible scenarios in the marine areas, there are river mouths, deltas, and wetlands, a wide range of values of salinity and turbidity are included for the calibration.

The calibration samples have a volume of 100 mL. Each was prepared in the laboratory using an analytical balance, NaCl, lime, and distilled water. NaCl was used as a source of dissolved solids which increased the salinity of the water. Meanwhile, the lime was the source of suspended solids.

The last variable parameter in our calibration is the temperature. The temperatures for the calibration were 14, 21 and 26 °C. The temperatures were attained by heating the water with a microwave and cooling the samples in the fridge. The temperature sensor, which is a commercial probe, was used to measure the temperature of the samples at the calibration moment.

### 4.2. Calibration Test

A calibration test is proposed to evaluate the suitability of the proposed sensor. Data from the different available sensors (inductive coils, IR photodiode, and LRD with the different lights) are gathered using the samples described above. The water of each sample is introduced into the methacrylate tube. One of the extremes of the tube was sealed for the calibration procedure. The sample was kept in the tube for 1 min in order to gather the calibration data. A total of five repetitions were performed.

### 4.3. Measurement Process

The sensor node is powered with the battery to gather the data of each calibration sample. When the node is powered, it starts to measure all the variables one by one. The node starts triggering the RGB LED and measuring the signal of the LDR. The light colours go from red, green, blue, yellow, purple, cyan, and white. One value of the LDR signal is gathered for each light colour. Then, the IR LED is turned on and data are collected from the IR photodiode. After that, the coil is powered, and the signal from an induced coil is measured. The whole process is entirely automatic and conducted in sequential order by the node. In order to avoid the impact of artificial light, which might affect the measurement procedure, a back box is used to cover the node.

The samples are carefully introduced into the tube and extracted using pipettes, since during the calibration the box is not waterproof. We start by measuring the samples with less salinity and TSS; sample 5. Then, the samples with the same TSS and higher salinity concentrations are used: samples 10, 15, 20, and 25. This way, we do not contaminate the salinity samples. After finishing measuring the samples with TSS equal to 0 mg/L, the tube is cleaned and the next set of samples is measured; the sample with TSS equals 9 mg/L, starting with sample 4, and then samples 9, 14, 19, and 24. This process is repeated, and the measurement finishes with samples 1, 6, 11, 16, and 21.

The node is connected to a computer to both power it and view the information. We have used the Arduino IDE monitor to see and gather the data in real-time. Data are obtained from the Arduino IDE monitor and introduced into Statgraphics for statistical analyses.

### 4.4. Statistical Analysis

Statgraphics Centurion XVIII is used for data processing. First of all, a multivariate analysis is carried out with all the data to evaluate if there are interferences and correlations between unexpected parameters. Then, single-way ANalysis Of Variance (ANOVA) and multiple groups tests were conducted for each correlated pair of variables between the measured parameter (salinity or TSS) and the sensor signal. Regarding the multiple group test, Duncan’s multiple range test is selected. Finally, for that cases in which correlation and ANOVA indicate that the measured parameter might be measured with the sensor, a regression model is obtained with Statgraphics. For the regression models, all available models for Stagraphics are considered, and based on the results of the comparison of alternative models the regression model with better R2 is selected. For all tests, 95% of confidence is used.

## 5. Results

In this section, we present and discuss our results. First, we show the general results of the calibration test. Then, the detailed data for the salinity are analysed. Subsequently, the evaluation of data from the TDS sensor is described. Finally, the main discussion linked to the obtained results is presented.

### 5.1. General Results of the Calibration Test

**Figure 7:**
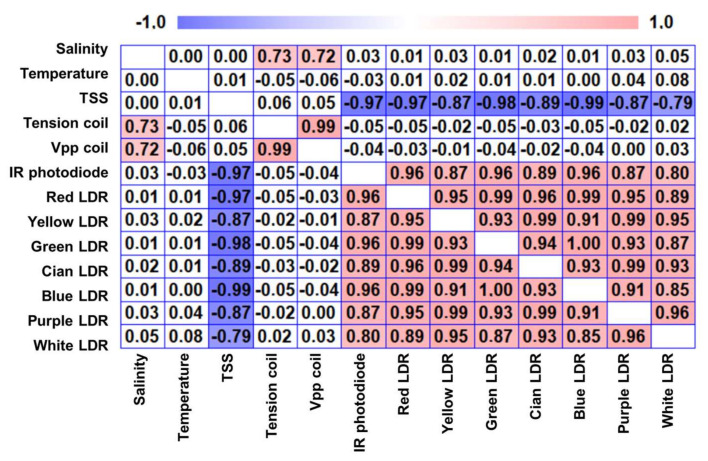
Correlation matrix from the multivariate analysis.

First of all, in order to evaluate if any of the target variables (temperature, salinity and TSS) might act as an interferent with the data gathering of another variable, a multivariate analysis is performed. The result of this analysis can be seen in Figure 7. The graphic depicts the correlation between included parameters and the measurement of the coil, the photodiode and the LDR with the different lights. The correlation values close to −1 and +1 indicate that the input variables are highly correlated. Meanwhile, the correlation values close to 0 indicate that variables are not correlated.

Focusing on the studied parameters, the temperature is not correlated with any of the sensors. Thus, it is possible to affirm that, for the tested temperature range (14 to 26 °C), the temperature does not act as an interferent for any of the measured variables (TSS and salinity) with the proposed sensors (electromagnetic and optical sensor). The salinity is correlated only with the response of the salinity sensor. This indicates that the salinity does not interact with the measurement of the TSS sensors. Regarding the TSS, it is only correlated with the measurement of the TSS sensors. In this case, the correlation is a strong negative correlation.

Concerning the response of the sensors, the tension coil and the Vpp coil (the input tension in the Arduino and the Vpp measured with an oscilloscope) only correlated between each other and with salinity. This indicates that any or both responses can be used for the calibration. The tension coil is preferred for the calibration since it is the input for the Arduino node. Therefore, the obtained calibration equation can be used directly in the node.

### 5.2. Calibration of Proposed Sensors

#### 5.2.1. Salinity Sensor

Before entering into detail, the results of a single way ANOVA are shown. The *p*-value of the ANOVA, equal to 0.000, indicated that the differences in Vpp of the coil for the different salinities are statistically significant. The multiple-group test revealed that four different groups could be identified in Table 2.

**Figure 8:**
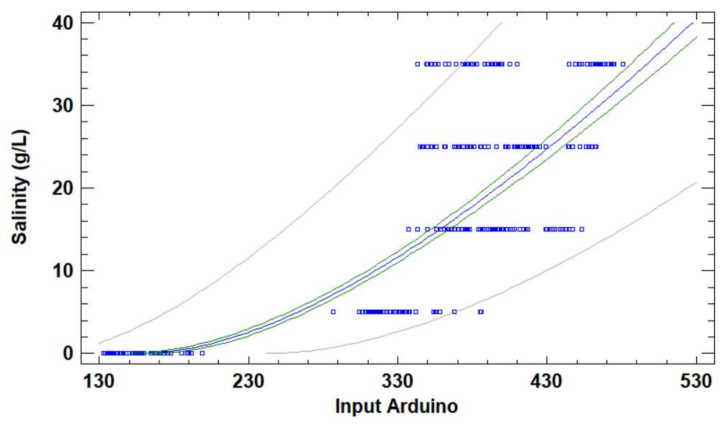
Calibration of the salinity sensor. Blue squares represent the calibration data. The blue line is the mathematical model, the green lines are the confidence interval, and the grey lines are the prediction interval.

A single regression model is used because the temperature and the turbidity were not correlated with the coil’s response in the previous subsection. All data obtained in the calibration test are used for the correlation. First of all, the detected outlier data are excluded from the linear regression. Figure 8 depicts the regression model for the salinity. The mathematical model, the equation to be used in the Arduino, can be seen in Equation (1). This model is characterised by a correlation coefficient of 0.90, a determination coefficient of 0.81, and an Absolute Mean Error (AME) of 0.74 g/L.

(1) $$Salinity\;\left(\frac{g}{L}\right)={\left(-7.5839+0.6055\times \sqrt{V_{Arduino}Coil}\right)}^{2}$$

where *Salinity* is the value of salinity in the calibration samples and the value of salinity in analysed new samples, and $V_{Arduino}Coil$ is the input analogue value in the pin in which the coil is connected to Arduino.

#### 5.2.2. TSS Sensor

As in the previous case, the first step is to analyse the results of the ANOVA. In this case, all the responses to different lights are included. The *p*-value of the ANOVA for all light sources is equal to 0.000, which indicates that the differences in the Arduino inputs for the different TSS are statistically significant. The multiple-group test revealed differences among the used lights. While with infrared, red, green, and blue lights it is possible to generate five different groups, for the rest of the lights only three groups can be identified. Thus, we use the lights that allow the generation of five groups for the obtention of calibration models.

**Figure 9:**
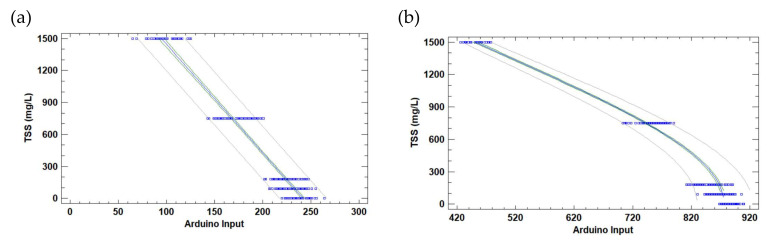

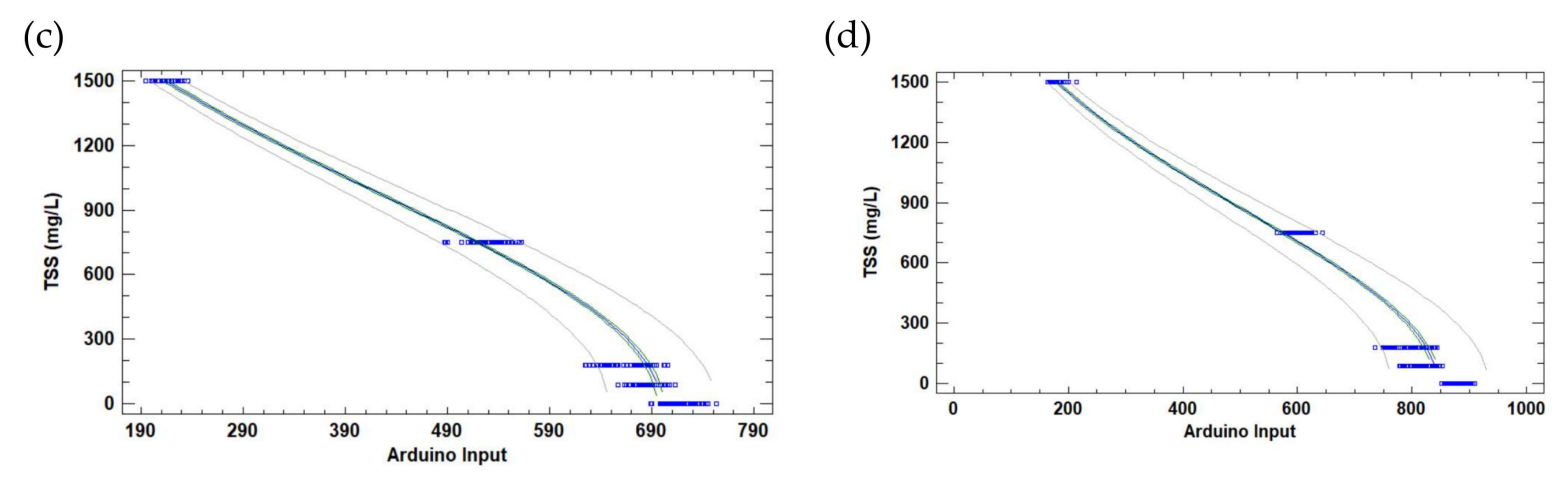
The calibration of the TSS sensor for different light sources: (**a**) IR light, (**b**) red light, (**c**) green light, and (**d**) blue light. Blue squares represent the calibration data. The blue line is the mathematical model, the green lines are the confidence interval, and the grey lines are the prediction interval.

As in the previous case, since there is no correlation between temperature or salinity with the light sensor responses, these data are not included in the regression models. All data obtained in the calibration test are used for the correlation. First of all, the detected outlier data are excluded from the analyses. Outliers are excluded individually for the dataset of each light source. Figure 9 depicts the individual regression models for the TSS and each light source. The mathematical models can be seen in Equations (2)–(5) for IR, red, green and blue lights. The models are characterised by correlation coefficients of a determination coefficient of −0.97 for the IR photodiode and −0.99 for the LDR, regardless of the used light. The determination coefficients are 0.94, 0.98, 0.99 and 0.99 for the IR photodiode, and LDR with red, green and blue light, respectively. The MAEs are 103, 31, 34, and 12 mg/L for each model.

(2) $$TSS\;\left(\frac{mg}{L}\right)=2463.35-10.179\times V_{Arduino}IR$$

(3) $$TSS\;\left(\frac{mg}{L}\right)=\sqrt{\left(2.3014\times 10^{7}-3.3967\times 10^{6}\times \ln(V_{Arduino}Red\right)}$$

(4) $$TSS\left(\frac{mg}{L}\right)=\sqrt{\left(1.248\times 10^{7}-1.9057\times 10^{6}\times \ln(V_{Arduino}Green\right)}\;\;$$

(5) $$TSS\;\left(\frac{mg}{L}\right)=\sqrt{\left(9.8241\times 10^{6}-1.4581\times 10^{6}\times \ln(V_{Arduino}Blue\right)}$$

where *TSS* is the value of TSS in the calibration samples and the value of TSS in analysed new samples, $V_{Arduino}IR$ is the input analogue value in the pin in which the IR photodiode is connected to Arduino, and $V_{Arduino}Red$, $V_{Arduino}Green$, and $V_{Arduino}Blue$ are the input analogue values in the pin where the LDR is connected to Arduino when different lights are used.

### 5.3. Measurement in Aquatic Systems

In order to validate the proposed system, measurements in a real aquatic system have been conducted. Two sites were selected for the verification of the system. The first site is an irrigation ditch, characterised by freshwater with high clarity and low TSS. The TSS of the irrigation ditch is mainly related to the degraded organic matter from the surrounding vegetation. It might be polluted by runoff from agricultural fields, which increases the turbidity and salinity and might even generate the eutrophication process. Nonetheless, at the measuring moment, no abnormal conditions were observed.

The second aquatic environment measured is the seashore, at the north side of the breakwater of the port. In this area, due to the predominant current and the sediment particle size in the beach, it is normal to find high turbidity values. The salinity of this area is not as high. At the moment of the data gathering, the water quality was not good. Over the previous days, a gale affected the area, and the wind and waves caused the movement of sediment, generating higher turbidity.

The data gathered by the sensors during the test can be seen in Table 3. It must be noted that, since TSS measurements require some time, three repetitions were performed and the mean value was used. We can see that the variation of gathered data from the optical sensors is lower for IR LED. Thus, we decided to apply the equations for the IR LED instead of using the model for the Blue LED.

After applying the equations described above, the TSS and salinity values for the seashore and the irrigation ditch water are the following: 3.39 and 227.33 mg/L of TSS and 0.37 and 34.20 g/L of salinity. These values are normal for these environments, especially considering the high turbidity found on the seashore due to inclement weather in previous days. Nonetheless, since most of the monitoring equipment is developed for measuring the turbidity in NTUs and the salinity as conductivity in mS/cm, a fair comparison cannot be made with existing probes. Even though it is possible to convert the salinity into mS/cm considering factors such as water temperature, the conversion of TSS in mg/L into NTUs depends on the type of particles causing the turbidity.

### 5.4. Discussion

#### 5.4.1. Analyses of Calibration Results

The salinity calibration showed that the proposed sensor could be used in coastal areas, mainly in regions with abrupt salinity changes, such as river mouths, areas with underground water emergence, or events in areas with emissaries or desalinisation plants [44,45]. It will be possible to detect the direction and magnitude of the freshwater or brackish water in the sea since in this case the variation in salinity is higher than 1 g/L. Nonetheless, with an MAE of 0.74 g/L and the reduced sensibility of the sensor for high values of salinity, the use of this sensor for monitoring the changes in salinity in very stable areas where the changes are less than 0.5 g/L this sensor is not recommended [46,47].

Concerning the TSS sensor, according to data for this calibration, blue light is the one that offered the most accurate measure of TSS. Nevertheless, according to several authors, IR light should be preferred. Therefore, we suggest creating a multiple regression model that incorporates both light sources in order to avoid and correct the effect of particle size. The multiple regression model obtained by combining both light sources is defined in Equation (6). It has a determination coefficient of 0.98 and an MAE of 57 mg/L.

(6) $$TSS\;\left(\frac{mg}{L}\right)=2104.65+1.7\times V_{Arduino}Blue+2.54\times V_{Arduino}IR$$

As for the salinity sensor, the MAE does not allow the direct use of this sensor for deep water or seawater characterisation. The typical TSS values in these waters are below 100 mg/L [48,49,50], and even below 10 mg/L in some areas [51].

Regarding the sensitivity of designed and tested sensors and existing ones, Table 4 summarises some examples. The sensitivity of our sensor has been calculated as the minimal detectable concentration according to the calibration curve and the node characteristics. After calculating the minimum detectable concentration, the sensitivity is converted into a percentage according to the minimum and maximum detected values. The proposed sensors’ sensitivity is similar to commercial probes and handheld equipment. The proposed system can be used as a probe to measure the environment, or as a handheld system with minor modifications in the code of the node.

Finally, considering the cost of commercial ready-to-use devices, the cost of our system is lower than the ones included in Table 5. The estimated price of the presented sensor node, including the sensors, the battery, the node, the SD card, the box, and all required circuits, is less than EUR 100.

#### 5.4.2. Potential Impact of the Proposed Multiparametric Probe

The low-cost, low-energy consumption, and low maintenance of the proposed multiparametric probe are aimed at maximising the possibility of its adoption. It is a low-maintenance probe, considering that no membranes, no electrodes, and no reagents are used for the water quality measurement. Even though cleaning will be necessary and strongly correlated with the monitored environment, the nutrients in the water, the epiphytes, and other fauna, this need will be lower for conductivity sensors based on electrodes. The correct design of the external protection and barriers against invertebrates and ichthyofauna, as well as deployment, ensures the avoidance of the obstruction of the tube for water measurement caused by sediments or fauna.

Under the project SALVADOR, part of the Thinkinblue program, we aim to promote water quality monitoring in coastal areas. This multiparametric probe is part of the proposed system composed of tens of probes deployed in different coastal areas, which send the gathered data to a database [61]. On the one hand, this generation of data will be crucial for local and regional marine managers, who will have updated data about the water quality of the coastal area. This will help them to improve the management of these areas. On the other hand, the data will also be accessible to the scientific community, who can use the data for the study of climate change, the recovery of natural areas, or changes in water due to pollution.

Moreover, according to the calibration, the proposed sensors of the multiparametric probe are not affected by temperature changes. This is relevant since it reduces the complexity of its operations, since no temperature regulation of correction for the data is needed, at least for the studied temperature range.

#### 5.4.3. Main Limitations of the Proposed Multiparametric Probe

The most relevant limitation is the relatively low sensibility of the probe for high salinity and low TSS values. These aspects will be considered and improved in future work, including new calibrations with more samples and physical and electronic changes to the physical sensors. Thus, the use of the multiparametric probe can be extended to other marine areas far from the coast.

On the other hand, more TSS than lime should be used, such as phytoplankton and organic matter. Using multiple light sources, it is most probable that the sensor can distinguish between different sources of TSS. Thus, including new calibrations, the sensor can provide data of TSS, turbidity in NTU, and chlorophyll-a in mg/m^3^, among others.

Regarding the node’s communication, the node is configured to operate with an ESP32 module using a WiFi connection. Nonetheless, the WiFi technology will only allow the monitorisation of superficial water. For monitoring the water column, the use of an Ethernet connection and the use of an additional ESP32 module on the water surface will be implemented. In these cases, as the box will be completely submerged under the water, it is recommended that the connection between them uses a low-losses wire such as the UTP 6e category to avoid problems in the data transmission. The ESP32 module will be located over the water surface inside a buoy, ensuring a good signal transmission over the air.

## 6. Conclusions

The need for low-cost and low-maintenance multiparametric probes is crucial for efficient coastal management. Nonetheless, the existing solutions are expensive and require considerable maintenance. This fact precludes the appropriate monitoring of coastal water. The most efficient option, the use of remote sensing, is limited in terms of temporal resolution and adverse climatological conditions.

In this paper, we have designed, created and calibrated a low-cost and low-maintenance multiparametric probe for coastal areas, composed of our own developed optical and electromagnetic sensors for salinity and TSS and a commercial probe for temperature. The scientific contribution of this paper can be summarised as follows:

- A sensor node combining two physical sensors (for TSS and for salinity) designed, created, calibrated in the laboratory, and verified in the real environment with a commercial probe for temperature has been presented.
- The complete design of the low-cost node is detailed, including the electronic components and the communication technology for integrating the node into a WSN.
- No electrical interferences of the sensors’ signal and circuits for powering the sensor and receiving the signal are detected when sensors are connected sequentially.
- No interference of salinity with the optical sensor for TSS measurement is detected.
- No interference of TSS in the electromagnetic sensor for salinity measurement is detected.
- No temperature effect on TSS and salinity sensors in the measured range is detected.

Future work will include the increment of calibration values to check if it is possible to improve the sensibility of the sensor at lower TSS values and higher salinity values. Moreover, if no improvement can be obtained, physical or electronic modification of the physical sensor will be considered. For the TSS sensor, the effect of increasing the tube size to have a greater optical path length and the reduction of light intensity on the sensibility for low TSS will be evaluated. For the salinity sensor, the increase of the water volume by using a tube with a bigger diameter increase the number of spires, and other coil configurations will be checked.

## Figures and Tables

**Table 1:** Samples for the salinity sensor calibration.

Sample ID	Added NaCl (g)	Added Lime (mg)	Salinity (g/L)	TDS (mg/L)
1	0	150	0	1500
2	0	75	0	750
3	0	18	0	180
4	0	9	0	90
5	0	0	0	0
6	0.5	150	5	1500
7	0.5	75	5	750
8	0.5	18	5	180
9	0.5	9	5	90
10	0.5	0	5	0
11	1.5	150	15	1500
12	1.5	75	15	750
13	1.5	18	15	180
14	1.5	9	15	90
15	1.5	0	15	0
16	2.5	150	25	1500
17	2.5	75	25	750
18	2.5	18	25	180
19	2.5	9	25	90
20	2.5	0	25	0
21	3.5	150	35	1500
22	3.5	75	35	750
23	3.5	18	35	180
24	3.5	9	35	90
25	3.5	0	35	0

**Table 2:** Summary of ANOVA for salinity.

Dependent Variable	Factor	F-Value	*p*-Value	Average VPP for				
				0 g/L	5 g/L	15 g/L	25 g/L	35 g/L
Tension Coil	Salinity	540.76	0.000	154.187 ^a^	333.613 ^b^	392.773 ^c^	401.267 ^cd^	408.24 ^d^

Different letters indicate different groups.

**Table 3:** Summary of ANOVAs for TSS.

Dependent Variable	Factor	F-Value	*p*-Value	Average Arduino Inputs for				
				0 mg/L	90 mg/L	180 mg/L	750 mg/L	1500 mg/L
IR photodiode	Turbidity	1669.45	0.000	238.52 ^e^	229.96 ^d^	224.04 ^c^	171.054 ^b^	93.6301 ^a^
Red LDR		3039.94	0.000	889.4 ^e^	867.64 ^d^	848.093 ^c^	751.419 ^b^	472.89 ^a^
Yellow LDR		1106.22	0.000	976.32 ^c^	973.76 ^c^	959.373 ^c^	957.176 ^b^	643.205 ^a^
Green LDR		4484.62	0.000	718.08 ^e^	683.907 ^d^	663.213 ^c^	525.811 ^b^	232.507 ^a^
Cian LDR		2855.08	0.000	973.893 ^c^	977.4 ^c^	969.307 ^c^	943.122 ^b^	386.685 ^a^
Blue LDR		**5533.26**	0.000	883.12 ^e^	813.293 ^d^	786.547 ^c^	584.311 ^b^	193.644 ^a^
Purple LDR		1256.16	0.000	976.467 ^c^	799.493 ^c^	972.307 ^c^	958.27 ^b^	616.767 ^a^
White LDR		293.22	0.000	977.97 ^bc^	980.107 ^c^	978 ^bc^	963.581 ^b^	771.767 ^a^

Different letters indicate different groups.

**Table 4:** Summary of data gathered in real tests.

Arduino Input for:	Irrigation Ditch				Seashore			
	Measure 1	Measure 2	Measure 3	Mean	Measure 1	Measure 2	Measure 3	Mean
Red LED	870	897	898	888.33	838	836	845	839.67
Green LED	667	751	732	716.67	613	656	684	651.00
Blue LED	875	874	891	880.00	702	796	808	768.67
Yellow LED	1003	961	964	976.00	956	945	977	959.33
Purple LED	967	988	951	968.67	964	975	974	971.00
Cian LED	956	1003	963	974.00	957	972	972	967.00
White LED	980	983	971	978.00	1000	966	964	976.67
IR LED	242	241	242	241.67	219	220	220	219.67
Inductive Coil	195	200	196	197.00	496	492	497	495.00

**Table 5:** Comparison with existing sensors in the market.

Ref.	Description	Measured Parameter	Measured Range	Sensitivity (%)	Price (EUR)
[52]	Handheld equipment	Turbidity (NTUs)	0–1000	2%	2813.00
[53]	Probe	Turbidity (NTUs)	0–4000	1%	N.I.
[54]	Handheld equipment	Turbidity (NTUs)	0–1000	2%	1670.71
[55]	Probe	Turbidity (FTUs)	0–1250	2%	N.I.
[56]	Handheld equipment	Turbidity (NTUs)	0–1100	2%	2020
Our	Handheld equipment and probe	TSS (mg/L)	0–1500	0.7%	<100
[57]	Handheld equipment	Conductivity (mS/cm)	0–400	1%	795
[58]	Probe	Salinity (ppt)	0–48	2%	979.00
[59]	Probe	Conductivity (mS/cm)	0–650	5%	N.I.
[60]	Probe	Conductivity (mS/cm)	0–200	N.I.	N.I.
Our	Handheld equipment and probe	Salinity (ppt)	0–35	0.94%	<100

## Data Availability

The data presented in this study are available on request from the corresponding author. The data are not publicly available due to privacy constraints.

## References

[1] Sunagawa S., Acinas S.G., Bork P., Bowler C., Babin M., Boss E., Cochrane G., de Vargas C., Follows M., Gorsky G. (2020). Tara Oceans: Towards global ocean ecosystems biology. Nat. Rev. Microbiol..

[2] Sala E., Mayorga J., Bradley D., Cabral R.B., Atwood T.B., Auber A., Cheung W., Costello C., Ferretti F., Friedlander A.M. (2021). Protecting the global ocean for biodiversity, food and climate. Nature.

[3] Gössling S., Hall C.M., Scott D. (2018). Coastal and ocean tourism. Handbook on Marine Environment Protection.

[4] Murray N.J., Phinn S.R., DeWitt M., Ferrari R., Johnston R., Lyons M.B., Clinton N., Thau D., Fuller R.A. (2019). The global distribution and trajectory of tidal flats. Nature.

[5] Danovaro R., Fanelli E., Aguzzi J., Billett D., Carugati L., Corinaldesi C., Dell’Anno A., Gjerde K., Jamieson A.J., Kark S. (2020). Ecological variables for developing a global deep-ocean monitoring and conservation strategy. Nat. Ecol. Evol..

[6] Bristow L.A., Mohr W., Ahmerkamp S., Kuypers M.M. (2017). Nutrients that limit growth in the ocean. Curr. Biol..

[7] Kundzewicz Z.W., Szwed M., Pińskwar I. (2019). Climate variability and floods—A global review. Water.

[8] Cardoso P., Barton P.S., Birkhofer K., Chichorro F., Deacon C., Fartmann T., Fukushima C.S., Gaigher R., Habel J.C., Hallmann C.A. (2020). Scientists’ warning to humanity on insect extinctions. Biol. Conserv..

[9] Watt A.J., Phillips M.R., Campbell C.A., Wells I., Hole S. (2019). Wireless Sensor Networks for monitoring underwater sediment transport. Sci. Total Environ..

[10] Jin S., Feng G.P., Gleason S. (2011). Remote sensing using GNSS signals: Current status and future directions. Adv. Space Res..

[11] Navalgund R.R., Jayaraman V., Roy P.S. (2007). Remote sensing applications: An overview. Curr. Sci..

[12] Zhang C., Marzougui A., Sankaran S. (2020). High-resolution satellite imagery applications in crop phenotyping: An overview. Comput. Electron. Agric..

[13] Hilker T., Wulder M.A., Coops N.C., Linke J., McDermid G., Masek J.G., Gao F., White J.C. (2009). A new data fusion model for high spatial-and temporal-resolution mapping of forest disturbance based on Landsat and MODIS. Remote Sens. Environ..

[14] Phiri D., Simwanda M., Salekin S., Nyirenda V.R., Murayama Y., Ranagalage M. (2020). Sentinel-2 data for land cover/use mapping: A review. Remote Sens..

[15] Alcantara C., Kuemmerle T., Prishchepov A.V., Radeloff V.C. (2012). Mapping abandoned agriculture with multi-temporal MODIS satellite data. Remote Sens. Environ..

[16] Bugalho L., Camara N., Kogan F. (2019). Study of wildfire environmental conditions in Portugal with NOAA/NESDIS satellite-based vegetation health index. J. Agric. Sci. Technol. B.

[17] Williams D.E. (2019). Low cost sensor networks: How do we know the data are reliable?. ACS Sens..

[18] Sendra S., Viciano-Tudela S., Ivars-Palomares A., Lloret J. Low-Cost Water Conductivity Sensor Based on a Parallel Plate Capacitor for Precision Agriculture. Proceedings of the International Conference on Advanced Intelligent Systems for Sustainable Development 2022.

[19] Jones S.B., Sheng W., Xu J., Robinson D.A. (2018). lectromagnetic sensors for water content: The need for international testing standards. Proceedings of the 2018 12th International Conference on Electromagnetic Wave Interaction with Water and Moist Substances (ISEMA).

[20] Maroni A., Tubaldi E., Ferguson N., Tarantino A., McDonald H., Zonta D. (2020). Electromagnetic sensors for underwater scour monitoring. Sensors.

[21] Yunus M.A.M., Mukhopadhyay S.C. (2010). Novel planar electromagnetic sensors for detection of nitrates and contamination in natural water sources. IEEE Sensors J..

[22] Nor A.S.M., Faramarzi M., Yunus M.A.M., Ibrahim S. (2014). Nitrate and sulfate estimations in water sources using a planar electromagnetic sensor array and artificial neural network method. IEEE Sensors J..

[23] Ahmad I., Ur Rehman M.M., Khan M., Abbas A., Ishfaq S., Malik S. (2019). Flow-based electromagnetic-type energy harvester using microplanar coil for IoT sensors application. Int. J. Energy Res..

[24] Das B., Jain P.C. (2017). Real-time water quality monitoring system using Internet of Things. Proceedings of the 2017 International Conference on Computer, Communications and Electronics (Comptelix).

[25] Parra L., Sendra S., Lloret J., Bosch I. (2015). Development of a conductivity sensor for monitoring groundwater resources to optimise water management in smart city environments. Sensors.

[26] Wang Y., Liu A., Han Y., Li T. (2020). Sensors based on conductive polymers and their composites: A review. Polym. Int..

[27] Azman A.A., Rahiman MH F., Taib M.N., Sidek N.H., Bakar IA A., Ali M.F. (2016). A low cost nephelometric turbidity sensor for continual domestic water quality monitoring system. Proceedings of the 2016 IEEE International Conference on Automatic Control and Intelligent Systems (I2CACIS).

[28] Arifin A., Irwan I., Abdullah B., Tahir D. (2017). Design of sensor water turbidity based on polymer optical fiber. Proceedings of the 2017 International Seminar on Sensors, Instrumentation, Measurement and Metrology (ISSIMM).

[29] Wang Y., Rajib SS M., Collins C., Grieve B. (2018). Low-cost turbidity sensor for low-power wireless monitoring of freshwater courses. IEEE Sensors J..

[30] Mulyana Y., Hakim D.L. (2018). Prototype of water turbidity monitoring system. IOP Conference Series: Materials Science and Engineering, Proceedings of the International Symposium on Materials and Electrical Engineering (ISMEE) 2017, Bandung, Indonesia, 16 November 2017.

[31] Parra L., Rocher J., Escrivá J., Lloret J. (2018). Design and development of low cost smart turbidity sensor for water quality monitoring in fish farms. Aquac. Eng..

[32] Mansor H., Maju NA H., Gunawan T.S., Ahmad R. (2022). The Development of Water Pollution Detector Using Conductivity And Turbidity Principles. IIUM Eng. J..

[33] Sharaf El Din E., Zhang Y., Suliman A. (2017). Mapping concentrations of surface water quality parameters using a novel remote sensing and artificial intelligence framework. Int. J. Remote Sens..

[34] Abdelmalik K.W. (2018). Role of statistical remote sensing for Inland water quality parameters prediction. Egypt. J. Remote Sens. Space Sci..

[35] Sagan V., Peterson K.T., Maimaitijiang M., Sidike P., Sloan J., Greeling B.A., Maalouf S., Adams C. (2020). Monitoring inland water quality using remote sensing: Potential and limitations of spectral indices, bio-optical simulations, machine learning, and cloud computing. Earth-Sci. Rev..

[36] Arduino Leonardo Module. https://www.farnell.com/datasheets/1682240.pdf.

[37] 7555 Integrated Circuit. https://www.analog.com/media/en/technical-documentation/data-sheets/icm7555-icm7556.pdf.

[38] Sendra S., Parra L., Ortuño V., Lloret J., De Valencia U.P. A low cost turbidity sensor development. Proceedings of the Seventh International Conference on Sensor Technologies and Applications (SENSORCOMM 2013).

[39] TSUS5400 Features. https://docs.rs-online.com/f3b6/0900766b80e22d5c.pdf.

[40] S186P Features. https://www.vishay.com/docs/81536/s186p.pdf.

[41] DS18B20 Temperature Sensor Features. https://www.analog.com/media/en/technical-documentation/data-sheets/ds18b20.pdf.

[42] DS1370 Features. https://www.analog.com/media/en/technical-documentation/data-sheets/ds1307.pdf.

[43] DS3231 Features. https://www.analog.com/media/en/technical-documentation/data-sheets/DS3231.pdf.

[44] Kress N., Gertner Y., Shoham-Frider E. (2020). Seawater quality at the brine discharge site from two mega size seawater reverse osmosis desalination plants in Israel (Eastern Mediterranean). Water Res..

[45] Fournier S., Lee T. (2021). Seasonal and interannual variability of sea surface salinity near major river mouths of the world ocean inferred from gridded satellite and in-situ salinity products. Remote Sens..

[46] Soto-Navarro J., Jordá G., Amores A., Cabos W., Somot S., Sevault F., Macías D., Djurdjevic V., Sannino G., Li L. (2020). Evolution of Mediterranean Sea water properties under climate change scenarios in the Med-CORDEX ensemble. Clim. Dyn..

[47] Serrano M.A., Cobos M., Magaña P.J., Díez-Minguito M. (2020). Sensitivity of Iberian estuaries to changes in sea water temperature, salinity, river flow, mean sea level, and tidal amplitudes. Estuar. Coast. Shelf Sci..

[48] Ashikur M.R., Rupom R.S., Sazzad M.H. (2021). A remote sensing approach to ascertain spatial and temporal variations of seawater quality parameters in the coastal area of Bay of Bengal, Bangladesh. Remote Sens. Appl. Soc. Environ..

[49] Bourouhou I., Salmoun F. (2021). Sea water quality monitoring using remote sensing techniques: A case study in Tangier-Ksar Sghir coastline. Environ. Monit. Assess..

[50] Bioresita F., Ummah M.H., Wulansari M., Putri N.A. (2021). Monitoring Seawater Quality in the Kali Porong Estuary as an Area for Lapindo Mud Disposal leveraging Google Earth Engine. IOP Conference Series: Earth and Environmental Science, Proceedings of the Geomatics International Conference 2021 (GEOICON 2021), Virtual, 27 July 2021.

[51] Patricio-Valerio L., Schroeder T., Devlin M.J., Qin Y., Smithers S. (2022). A Machine Learning Algorithm for Himawari-8 Total Suspended Solids Retrievals in the Great Barrier Reef. Remote Sens..

[52] 2100Q Portable Turbidimeter from Hach. https://uk.hach.com/turbidimeters/2100q-portable-turbidimeter/family?productCategoryId=25046201232.

[53] Solitax t-Line sc Turbidity Immersion probe from Hach. https://www.hach.com/p-solitax-t-line-sc-turbidity-immersion-probe-0001-4000-ntu-with-wiper-pvc/LXV423.99.10000.

[54] HI-98703 Turbidity Meter from Hanna Instruments. https://www.hannainstruments.co.uk/home/1818-turbidity-meter.

[55] Seapoint Turbidity Meter. http://www.seapoint.com/stm.htm.

[56] Turbiditymeter from TURBIQUANT. https://www.sigmaaldrich.com/ES/es/product/mm/118325?gclid=CjwKCAiAzp6eBhByEiwA_gGq5KByrMzJ-HgVxVXfT-eW_PVxKUuEX2kpksGOLuli-CYxW9t7VgbjAxoC1AkQAvD_BwE&gclsrc=aw.ds.

[57] HI-98192 from Hanna Instruments. https://www.hannainstruments.co.uk/multi-parameter-devices/2131-hi-98192-professional-waterproof-ec-tds-resistivity-salinity-meter.

[58] Intellical CDC401 Field from Hach. https://uk.hach.com/intellical-cdc401-field-4-poles-graphite-conductivity-cell-5-m-cable/product?id=24929274325&callback=qs.

[59] InPro7108-VP-PEEK from Mettler Toledo. https://www.mt.com/es/en/home/products/Process-Analytics/conductivity-resistivity-analyzers/conductivity-sensor/probe-InPro-7108-VP-PEEK.html#documents.

[60] Orion™ DuraProbe™ from Thermo Fisher Scientific. https://www.thermofisher.com/order/catalog/product/013010MD.

[61] Sendra S., Parra L., Jimenez J.M., Garcia L., Lloret J. (2022). LoRa-based network for water quality monitoring in coastal areas. Mob. Netw. Appl..

